# An Adaptive Method for Switching between Pedestrian/Car Indoor Positioning Algorithms based on Multilayer Time Sequences

**DOI:** 10.3390/s18030711

**Published:** 2018-02-27

**Authors:** Zhining Gu, Wei Guo, Chaoyang Li, Xinyan Zhu, Tao Guo

**Affiliations:** 1 State Key Laboratory of Information Engineering in Surveying, Mapping and Remote Sensing, Wuhan University, Wuhan 430072, China; zhininggu@whu.edu.cn (Z.G.); xinyanzhu@whu.edu.cn (X.Z.); 2 National Network SiJiShenWang Location Service (Beijing) Co., Ltd., Beijing 102200, China; clsdcn@163.com; 3 Collaborative Innovation Center of Geospatial Technology, Wuhan University, 129 Luoyu Road, Wuhan 430079, China; 4 Wuhan Digital Engineering Research Institute, No. 718, Luoyu Road, Hongshan District, Wuhan 430000, China; guotao32@126.com

**Keywords:** behavior context, MTS, state recognition, switching pedestrian/car positioning algorithm

## Abstract

Pedestrian dead reckoning (PDR) positioning algorithms can be used to obtain a target’s location only for movement with step features and not for driving, for which the trilateral Bluetooth indoor positioning method can be used. In this study, to obtain the precise locations of different states (pedestrian/car) using the corresponding positioning algorithms, we propose an adaptive method for switching between the PDR and car indoor positioning algorithms based on multilayer time sequences (MTSs). MTSs, which consider the behavior context, comprise two main aspects: filtering of noisy data in small-scale time sequences and using a state chain to reduce the time delay of algorithm switching in large-scale time sequences. The proposed method can be expected to realize the recognition of stationary, walking, driving, or other states; switch to the correct indoor positioning algorithm; and improve the accuracy of localization compared to using a single positioning algorithm. Our experiments show that the recognition of static, walking, driving, and other states improves by 5.5%, 45.47%, 26.23%, and 21% on average, respectively, compared with convolutional neural network (CNN) method. The time delay decreases by approximately 0.5–8.5 s for the transition between states and by approximately 24 s for the entire process.

## 1. Introduction

In recent years, the importance of mobile devices, particularly smartphones, has increased for indoor positioning. The widespread use of these devices enables location-based services (LBSs) to be easily made available [1] for every user. Moreover, this makes indoor positioning and navigation more realizable. Accurate user localization, which is regarded as an essential component of LBSs, can be provided using the embedded sensors and modules of smartphones [1]. The inner sensors of mobile phones are used widely to assist with localization in indoor parking, as they overcome the problem of weak signals in indoor environments and provide a convenient method for users to park. Numerous indoor positioning methods are being developed for use inside covered structures. Wi-Fi, Radio Frequency Identification (RFID), Bluetooth, Ultra Wideband (UWB), pedestrian dead reckoning (PDR), and a few other technologies are widely used on the basis of existing deployment environments or different targets [2]. Current indoor positioning approaches are already quite mature owing to these technologies. 

There are some methods which are popularly used for positioning in indoor environments such as parking lots, with different principles [3,4,5]. The Wi-Fi-based indoor positioning method takes advantage of received signal strength, but usually, Wi-Fi is not available in parking areas and other facilities on which this work focuses. On the other hand, Bluetooth can offer better indoor positioning accuracy than Wi-Fi AP’s [6,7]. The fingerprint-based indoor positioning method comprises offline training and online testing to realize localization [8,9,10], but it needs a lot of computation and some other resource consumption. IBeacon is a Bluetooth signal source. Targets can receive the signals emitted by IBeacons through smartphones [8,11,12] to locate their positions. All these signals-of-opportunity approaches are available as infrastructure and can be utilized. Among these various methods, IBeacons are easier to be deployed with low cost, and can be used for many years due to low power consumption [13,14]. Compared with other position approaches [15], it’s more convenient, cheaper and lower power [16]. However, almost all methods used in indoor positioning for obtaining highly precise location results depend on existing deployment environments and do not use the context information generated by the movement of targets such as users or mobile phones [17]. Furthermore, several states exist in indoor environments, such as driving, stationary, and walking. One positioning method cannot be used for all states. The PDR algorithm is frequently used for the pedestrian state; the algorithm can be used for movement with the step feature and not for driving, and it estimates the movement of pedestrians on the basis of the sensors (accelerometer, gyroscope, and magnetometer) embedded in a smartphone [18]. This type of method can reduce the cost, power consumption, and computational resources and utilize the modules in smartphones. For example, taking car positioning in parking lots, the driving state typically occurs on roads and effectively removes one dimension from the position solution [19]. Thus, it lacks the periodic pedestrian accelerations during movement. The PDR positioning algorithm cannot be used in this case, and positioning can only be achieved based on WiFi, Bluetooth or some other technology. There is no single navigation positioning technology that can be used in all situations. From this viewpoint, a new robust positioning switching method that is suitable for different situations such as the pedestrian or driving states should be developed with environmental feature matching and context detection methods to obtain a reliable, cost-effective, and integrated system [20]. Therefore, we choose PDR for detecting pedestrians’ movement, and IBeacons for cars movement. These two algorithms’ combination can reduce some unnecessary computation and power consumption according to the corresponding states.

The basis of the switching algorithm is recognition [21]. Previous work on recognition has focused on the sensors embedded in mobile devices, particularly smartphones, which is a popular research topic [22]. The data obtained from these smartphone sensors can be used as context to provide useful and relevant information or services to users [23]. The motions involved in daily activities, such as walking, jogging, sitting, and running, are more complicated on account of arbitrary gestures [24]. MEMS inertial sensors are attached to humans, and an inference model can be trained according to a few body positions [25,26]. The aim of activity recognition algorithms is to obtain information about the activity of a user [24]. Altun et al. classified human activities using body-worn inertial and magnetic sensors [17,23]. The results showed reasonable accuracy, however, individual adaptation is necessary for applications that require extra context information. In addition, extensive computation is required to recognize the current state in real-time. Gu et al. introduced several classification and automatic feature selection algorithms [23,27]. They focused on common daily activities that collaboratively or concurrently involve multiple users and presented the first formal study on using a body sensor network for multiuser activity recognition. However, the design of their sensor network is still premature and a long way from real-life deployment [27].

Human activity recognition is a significant research field with several challenges, most of which focus on the accuracy, robustness, and real-time capability [28]. Machine learning is a well-known approach for recognition [29]. Numerous related works have used machine learning to design, develop, and evaluate the capability of learning from sensor data and have demonstrated its learning capacity and encouraging performance in widespread fields. Data are used as the input of learning processes [7]. Recognition results are the output after passing through a trained model. A number of methods can be applied to recognition. Chen and Xue proposed a convolutional neural network (CNN) approach for recognition, which can extract features and does not require any domain-specific knowledge about acceleration data [2,30]. Lee and Yoon proposed a method of constructing a novel activity image for deep CNNs using gyroscope and acceleration signals [31]. The method exhibited better performance than the feature selection method [31]. In addition to the CNN approach, there are other methods such as support vector machines (SVMs), decision trees, Bayesian methods, neural networks, logistic regression, and hidden Markov models [2,32]. However, most of these methods mainly describe how to use machine learning to recognize a single state such as walking or jogging. The following challenges still remain:

(a) All these approaches are based on single-state identification (monomer identification), for which the accuracy is high. However, under natural conditions, there are numerous complex and diverse continuous states, which should be recognized precisely. Therefore, in this case, CNN recognition cannot satisfy the requirements of preciseness, even though it can achieve high-accuracy results for a single state.
(b) Under natural conditions, there is a large amount of noisy data because certain correct states can be recognized as other false states in sophisticated indoor environments. Noisy data must be removed on the basis of recognition.
(c) The methods that use CNNs utilize the accelerometer data in the sensors embedded in smartphones and neglect the features of moving targets [33,34,35]. This leads to failure in differentiating between similar states. For example, the stationary state and the driving state with a constant speed are difficult to recognize if the data from sensors are used. However, these states are significantly different in terms of the movement velocity, which can be obtained from an indoor positioning scene based on the features of moving objects. Therefore, we require the movement features of targets in indoor positioning environments to assist with the switching of algorithms during recognition.

**Figure 1:**
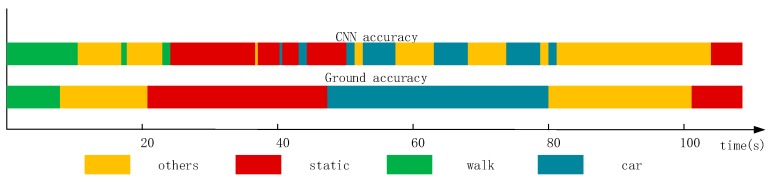
Recognition results obtained using the CNN method. The upper band called CNN accuracy is the result based on CNN method. The lower band named ground accuracy is the real movement results as a reference of CNN accuracy.

Figure 1 shows that there is a large amount of noisy data when the CNN method is used in a natural environment. The states are not easy to distinguish. As it shown, there are two bands: the upper band named CNN accuracy and the lower band called ground accuracy. The ground accuracy is the authentic movement results in natural environment and can be considered as the reference of CNN accuracy results which are based on CNN method. From this figure, we can see that there is a large amount of noisy data. For example, during the 60 s–80 s, the real state of user’s movement was “car” (driving), which can be seen in the ground accuracy band. However, when we used CNN method to recognize the current state which should have been shown the color of “car”, it generated a lot of some “others” states (yellow color) which we call noisy data.

To solve these problems, in this paper we propose an adaptive method for switching between indoor positioning algorithms. This method is based on current conventional indoor positioning technology and the sensors embedded in smartphones. We use the data collected from these sensors to obtain the related behavior information to recognize if the current state is the pedestrian or driving state and to switch to the correct algorithm accordingly. By using the algorithm that corresponds to the current state, more accurate positioning can be obtained. Therefore, we mainly aim to recognize the current state of users under indoor localization conditions to realize this method for improving position technology.

The rest of this paper is organized as follows: Section 2 presents the proposed adaptive method for switching between positioning algorithms. In Section 3, our results are compared with the results of other methods. Finally, the conclusions and the plans for future work are presented in Section 4.

## 2. Methods 

To overcome the abovementioned challenges, we propose an adaptive indoor algorithm switching method based on a CNN using four states: static, vehicle, pedestrian, and “other”. If a user uses a smartphone, his/her speed under any type of these four states can be obtained. We can get his/her Bluetooth speed by using a Bluetooth indoor positioning method. His/her PDR speed also can be obtained by taking advantage of the accelerometer and gyroscope in the smartphone. For these two kinds of speed, they will show different features in different states. Here, for a static state, both of them should theoretically give a value of 0 or close to 0. However, because the embedded sensors, mainly including accelerometers and gyroscopes, are so sensitive, the PDR speed can be influenced by tiny quivering of targets, even if their positions have not changed. Second, for vehicle states, which we also can call driving or car, we consider it as a vehicle state when the car is driven speeding up or slowing down, but not at constant speed. If car is travelling at a constant speed, a smartphone with embedded sensors as modules placed in the car will detect a stationary state through these sensors. At this time, the real state of the target, namely the smartphone or some other device with embedded sensors, is stationary even though the car is moving. For convenience in our further study, we can classify this state as a stationary state as a first approach, and in the following process, we will decide whether it’s really a stationary or an in-car state depending on the velocity. As for the pedestrian state, we consider it as walking. Of course in an indoor environment, people can run or walk faster than a car, so here we include these as walking states for further processing convenience. In addition, it has step detection features, so the PDR and Bluetooth velocity should be similar theoretically and they will be stable around a certain speed when walking, but for other stats, they don’t have similar features. Therefore, we can differentiate walking from the others depending on the speed. Fourth, we consider when users shake their phones or some other random vibrations which do not generate any position changes as the “other” states. They can be that phones are put in bags or held in hands with shaking. For example, a user just stands in a spot, shaking his/her phone. In this kind of situation, there is no position change, which means the Bluetooth velocity will be 0 or closed to 0 in theory, but the acceleration values will change dramatically and with no regularity. The entire process is based on the data obtained from the sensors embedded in smartphones and the indoor positioning scene information which are related with users’ current state (stationary, in-car, walking, others) by using of PDR and Bluetooth velocity of users with portable smartphones [22,36]. The CNN method is adopted owing to its superiority for extracting features without any domain-specific knowledge about the acceleration data. In terms of the indoor positioning information, the velocity is different among the states; hence, we utilize Bluetooth positioning results, which are trained by an SVM, to assist users in determining their current state. The main contributions of this method are as follows:

(a) First, a well-known deep learning method is used to train the state data. It extracts features without any domain-specific knowledge about the acceleration data.
(b) Second, we propose a multilayer time sequence (MTS) method that combines the positioning information of an indoor environment to improve the accuracy of recognition or to remove noise and switch to the correct algorithm according to the current state. This method can consider the unique features of each state.
(c) Third, our method can reduce the cost, power consumption, and computational resources because all algorithms do not require extensive computation or a high power. The power consumption can be reduced if one algorithm is switched to another.

**Figure 2:**
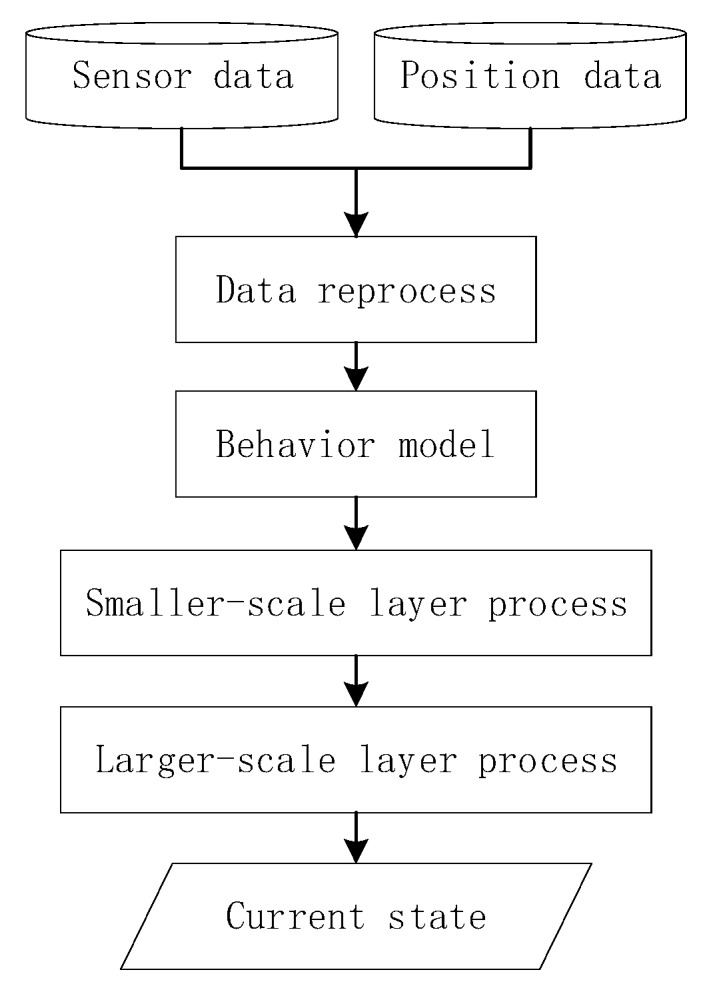
Overall flowchart of proposed method.

As shown in Figure 2, data are obtained from mobile phones and reprocessed in real-time. On the basis of the behavior-trained models developed using the deep learning method, we propose an MTS approach that provides two channels of abstraction. These channels enable users to reach their desired target. First, we describe a smaller scale that uses sliding time plots to remove noisy data according to the priority of the results trained from the sensors and indoor positioning technology. Second, on the basis of the results obtained from the first channel, we present a state chain method in a larger-layer to filter noisy data more extensively and to reduce the time delay when switching to another algorithm. As the final aim of recognition is to switch to the appropriate algorithm, the time delay of switching into the current state should be considered. Therefore, in this section, we first define the state codes and state events to comprehend the method more conveniently. Then, we describe the proposed method in more detail by dividing it into the following three parts: priority strategy, noise filtering with fine granularity (a smaller-scale), and optimization based on a state chain in a higher layer (a larger-scale).

### 2.1. Definition

In this section, we define two terms used to describe the MTS method, i.e., the state codes and state events. The state codes are the results based on two types of training models. The first is expressed as harStateCode; it is obtained from the CNN method using the acceleration of the inner sensors. The second is generated by the positioning information of targets using SVM training; it is referred to as svmStateCode. A state event is a basic component of a series of continuous states; it is expressed in the “end-to-end” form, which contains the states that generate movement at the start and end.

#### 2.1.1. State Codes

We primarily use state codes as processed data to realize the MTS method. They contain the following two parts: the state codes obtained from CNN results and the state codes generated by the SVM in terms of the velocity. In this study, we select four basic human daily behavior modes: stationary, walking, in car, and others. They are represented into St, Wa, Ca and Ot, respectively. For the following explanation, we will discuss two different situations using these four states. One is the state that doesn’t generate any changes of positioning (St, Ot), another is that location will change (Ca, Wa). Therefore, in order to describe more conveniently, we define these basic modes as number codes as follows:

(1) BS=MS∪SS,

MS={Ca, Wa}, SS={St, Ot},

where *MS* is a collection of basic modes in which people change their position; it includes *Ca* (in car) and *Wa* (walking). *SS* consists of *St* (stationary) and *Ot* (others) without movement. *BS* is a collection of basic human behavior modes consisting of *St*, *Ca*, *Wa*, and *Ot*. We use the position indices of the state codes as their values for convenient processing. For example, the state code value of *St* is 1 because its index is 1. Therefore, *Ca* is 2, *Wa* is 3, and *Ot* is 4.

The first part of the state codes is generated from the CNN results. We adopt CNNs to train the acceleration data obtained from sensors. A CNN can extract the feature values from these sensor data automatically, preventing artificial extraction. The use of a CNN approach can be more efficiently and accurately be implemented and reduces human effort. Furthermore, the X/Y/Z acceleration data can be regarded as three channels of images, i.e., the length, width, and height. The data we collect are continuous. Thus, we can utilize a CNN thanks to its advantages to resolve our problem, considering the three-axis data as images to train an appropriate model. We design the CNN with an input layer, two convolutional layers, two max pooling layers, a fully connected layer, and an output layer [3,24]. On the basis of the trained model, we can obtain a human activity in real-time:

(2) $$f_{cnn}\left(\omega ,\;b\right)\to harStateCode\;\left(harStateCode\in BS\right),$$

where ω and *b* denote the weight and bias in the trained CNN model, respectively. We use $f_{cnn}$ to calculate and recognize the current behavior activity, namely, harStateCode. harStateCode should belong to one of the *BS* collections (stationary, in car, walking, or “other”).

The second part of the state codes is generated from the SVM results. We use the PDR velocity, $v_{pdr}$, and the Bluetooth velocity of an user who takes a portable smartphone in trilateral Bluetooth positioning, $v_{tri}$. Here, we adopted IBeacon to deploy our experiment environment due to its low power consumption with lower cost. And we also define the velocity of a target in the form ($v_{pdr},v_{tri}$). We employ these two categories because PDR positioning is based on step detection, step-length estimation, and heading estimation. This velocity is closer to the speed of a user when they are walking. The other states (such as the driving state) cannot adapt to this walking situation well, in that the velocities are completely different and the velocity while driving is greater than that during walking. More information about other states can be obtained using a few features of the Bluetooth velocity. For instance, when a user is stationary, his/her velocity is extremely small and close to (0, 0). When a user is walking, because of the accuracy of PDR and the trilateral positioning, the velocity is maintained within a certain range and is lower than the speed of a car. Moreover, these two velocities are considerably close under conditions where the PDR and trilateral positioning results are ensured. PDR cannot be applied precisely to the in-car state owing to the lack of the characteristic steps, however, trilateral Bluetooth positioning provides accurate results in this case. Thus, $v_{pdr}$ does not exhibit regularity, but $v_{tri}$ is comparatively stable and higher. For the other states, $v_{pdr}$ and $v_{tri}$ are messy and have no regularity. This can be used to classify the states. Furthermore, to classify the four states, at least two eigenvalues are required to separate them. According to the trained SVM model, we can obtain the current state as:

(3) $$f_{svm}\left(\omega^{*},\;b^{*}\right)\to svmStateCode\left(svmStateCode\in BS\right),$$

where $\omega^{*}$ and $b^{*}$ are the weight and bias in the trained SVM model, respectively. We use $f_{svm}$ to calculate and recognize the current behavior activity in terms of the velocity, namely, svmStateCode. svmStateCode should belong to one of the BS collections.

The two main state code values (harStateCode and svmStateCode) can be used for further processing to obtain the final state of the target as follows:

(4) g(harStateCode, svmStateCode)→finalStateCode(finalStateCode∈BS),

where g(harStateCode, svmStateCode) uses (harStateCode, svmStateCode) as the input for the MTS method. finalStateCode is the final state of the target as the output.

#### 2.1.2. State Event

Targets have flexible motions indoors; they can change their states. Our aim is to recognize the current state of a target and switch to the appropriate algorithm when the positioning of the target changes; that is, the corresponding positioning algorithm can be triggered only when a target’s location changes. When a user is in the stationary state, his/her position does not change; thus, there is no need to use a positioning algorithm to update the localization. For the walking state, the algorithm should be switched to PDR positioning in real-time. The trilateral Bluetooth positioning algorithm can be used in the in-car state.

This study considered four behavior states; however, only the walking and in-car states cause a change in localization. Among them, we assume that the target motion state is continuous rather than a variety of simultaneous states. Therefore, we can regard all movements as a combination of these basic states and trigger the corresponding positioning algorithm in the walking or in-car states. Here, we define an end-to-end “state event” as a basic unit that contains the state that leads to a change in the position. For example, for a state segment, the start and end points can be the walking or in-car states, in which a user moves. Between the start and the end, the middle states should be the stationary state or other states; in this case, the target is still at the same location.

**Figure 3:**
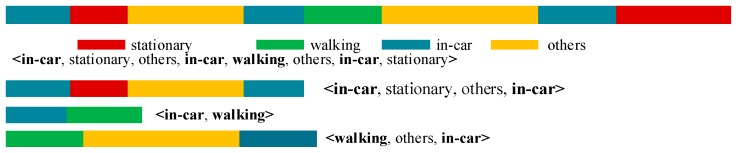
Example of a state event.

Figure 3 shows an example in which it is assumed that a user performs certain actions (in-car → stationary → others → in-car → walking → others → in-car → stationary). These types of continuous states can be considered as a combination of a few end-to-end basic events. That is, (in-car, stationary, others, in-car, walking, others, in-car, stationary) can be expressed as <in-car, …, in-car>, <in-car, walking>, and <walking, …, in-car>. Each component contains the in-car or walking state at the start and end, and the stationary and other states are between them.

Therefore, we define a state event SE as follows:

(5) $$SE=\cup_{i=1}^{N}{\langle state_{start}^{i}\;:\;state_{end}^{i}\rangle }_{i}\left(state_{start}^{i}:state_{end}^{i}\;\in BS\backslash SS\right),$$

(6) $$\langle state_{start}^{i}\;:\;state_{end}^{i}\rangle =\;\left(state_{start}^{i},state_{start+1}^{i}\right)\cup \left(state_{end-1}^{i},\;state_{end}^{i}\right)$$

where SE is a series of continuous movements in a period. It contains N end-to-end basic state events, indicated as ${\langle state_{start}^{i}\;:\;state_{end}^{i}\rangle }_{i}$. In this expression, the start and end points belong to BS\SS, namely, {Ca, Wa}, but among it there are still several states which belong to MS. So we use Equation (6) to divide the whole process into several segments shown in the right part whose MS states are only in the start and end. There are numerous state events; however, there are only a few end-to-end basic state events, as listed in Table 1.

Considering Figure 3 as an example, a state event can be expressed as $SE=\;\cup_{i=1}^{3}{\langle state_{start}^{i}\;:\;state_{end}^{i}\rangle }_{i}={\langle Ca:Ca\rangle }_{1}\cup {\langle Ca:Wa\rangle }_{2}\cup {\langle Wa:Ca\rangle }_{3}$. For ${\langle Ca:Ca\rangle }_{1}$, this is equal to ${\left(Ca,St\right)}_{1}\cup {\left(St,Ot\right)}_{1}\cup {\left(Ot,Ca\right)}_{1}$. Similarly, ${\langle Ca:Wa\rangle }_{2}\;={\left(Ca,Wa\right)}_{1}$ and ${\langle Wa:Ca\rangle }_{3}={\left(Wa,Ot\right)}_{3}\cup {\left(Ot,Ca\right)}_{3}$.

### 2.2. MTS Process Based on Behavior Context

On the basis of the abovementioned definitions, we propose a processing method using MTSs based on the behavior context. The MTS process contains two components. The first is noise filtering in a lower layer with sliding time plots. The second is optimization using the state chain proposed in a higher layer with a given behavior context (harStateCode, svmStateCode) from the first channel to filter more extensively and reduce the time delay. First, we discuss the priority strategy, which contributes to the priority of harStateCode and svmStateCode. In this section, we consider the MTS process based on three aspects: priority strategy, noise filtering with fine granularity, and optimization based on the state chain proposed with a coarse time granularity.

#### 2.2.1. Priority Strategy

According to the abovementioned descriptions of svmStateCode and harStateCode, they are the results of human activity recognition. Given these two types of results, we select one of them as the final behaviors of users, namely, finalStateCode. These states have their own different characteristics in the aspect of PDR velocity and Bluetooth velocity. For the stationary state, the speed of PDR and Bluetooth is 0 or close to 0. Cars do not have any feature in PDR due to their lack of step features, but have high or low speed. Pedestrians have both. For “other” states, the results of Bluetooth will be close to 0 and they don’t have PDR features when shaking or vibrating. Because the accelerometer and gyroscope can detect velocity changes in these conditions, these velocities can be very high at some time, and become slow at other times. A PDR velocity without any regularity will exist. Based on the above, if svmStateCode and harStateCode are inconsistent, we select one of them as the final behavior result or use a process to obtain the result in accordance with the actual movement state of a user based on these features in PDR velocity and Bluetooth velocity in order to preprocess the data and make a forecast. Therefore, on this basis, we propose the priority strategy concept for determining which of the two state codes occupies a larger proportion.

We consider that svmStateCode and harStateCode have the same priority when their values are equal. In the next step, we can use their code values as the final behavior results. However, when the values are not equal, the priorities are determined by the behavior context. That is, if a svmStateCode’s characteristic is closer to a certain feature of PDR and Bluetooth velocity than harStateCod, we consider it’s more suitable for the current behavior context than harStateCode, then svmStateCode is more preferred than harStateCode. Note that we only consider the priorities as a judgement and do not depend entirely on the priorities in the final state. In the next step of noise filtering and optimization, we use the priorities as a basis for processing in complicated and ambiguous situations.

#### 2.2.2. Noise Filtering with Fine Granularity

When people move, the mobile phones carried by them can detect their behavior in real-time to output a series of svmStateCode and harStateCode results. These results are not exactly the same as the actual movement state. Incorrect state data always exist. Thus, noisy data should be removed to obtain continuous correct data according to the situation. We propose a noise filtering method based on fine granularity.

**Figure 4:**
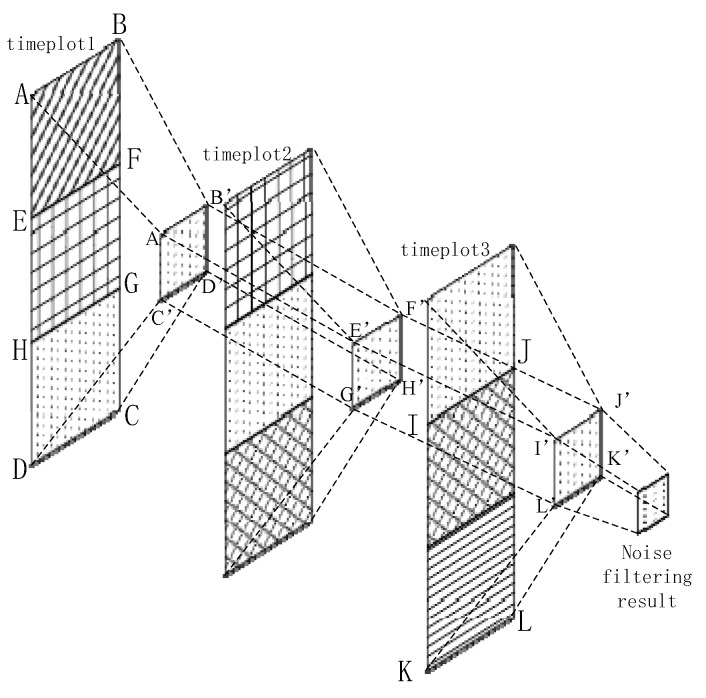
Noise filtering model.

We generate three continuous time plots based on the proposed approach, each with a fixed width of approximately 3 s. The overlap between two adjacent periods is 1 s. We can use Figure 4 to express the noise filtering method using time plots abstractly. In Figure 4, we regard each rectangle as states of 1 second. For example, rectangle ABFE contains the harStateCode and svmStateCode for first one second. Rectangle EFGH stands for the states for second one second. These rectangles with the same texture in different time plots are the same one. In other words, the second rectangle of timeplot1 is the first one in timeplot2. The third one of timeplot1 is the second one of timeplot2, etc. Each rectangle with own texture represents corresponding state codes for 1 second. The time interval of each adjacent rectangle (timeplot1 & timeplot2, or timeplot2 & timeplot3) is one second. We mainly process the 1 s time sequence with the maximum overlap, namely rectangle HGCD which exists in all of time plots. Among the figure, rectangle ABGH can be regarded as the a priori behavior context, and rectangle IJKL can be seen as the posterior behavior context. Our method extracts the maximum voting values from each time plot, which means the value exists in current time plot with most frequency in a certain time plot and are represented into rectangle A’B’D’C’, E’F’H’G’ and I’J’K’L’. Then we use them to obtain the final noise filtering result. 

Therefore, on the basis of the current context with the a priori context and posteriori context, we can obtain the noise filtering result of the current context, which means the generation of wrong states in real state, just like the description of Figure 1. We use the majority voting rule based on the a priori context and posterior context. First, we obtain the state codes from svmStateCode and harStateCode. If they are equivalent, then the state label is svmStateCode or harStateCode; both have the same priority. Otherwise, we mark the state label as 0, and its priority is determined by the a priori context and posterior context as:

(7) $$StateLabel=\{\begin{array}{c} svmStateCode,\;\left(svmStateCode=harStateCode\right)\\ \;\;\;\;\;\;0\;\;\;\;\;\;,\;\left(svmStateCode\;!=harStateCode\right) \end{array},\;\;svmStateCode,harStateCode\in BS.$$

**Figure 5:**
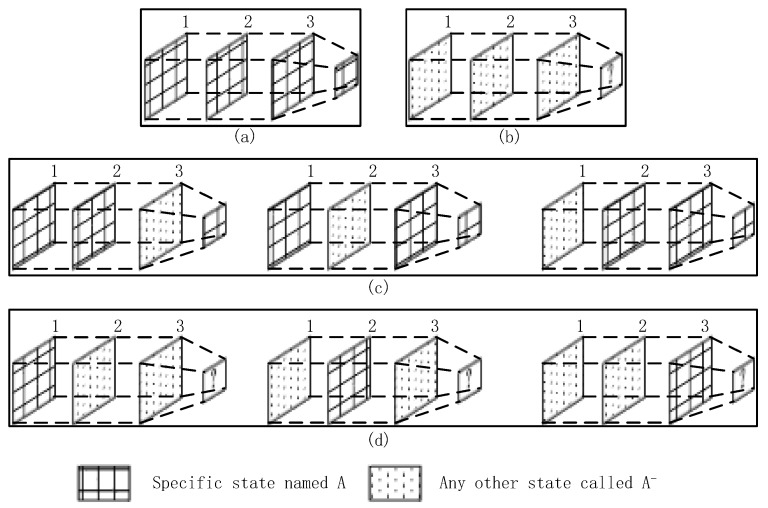
Circumstances of a certain state.

**Figure 6:**
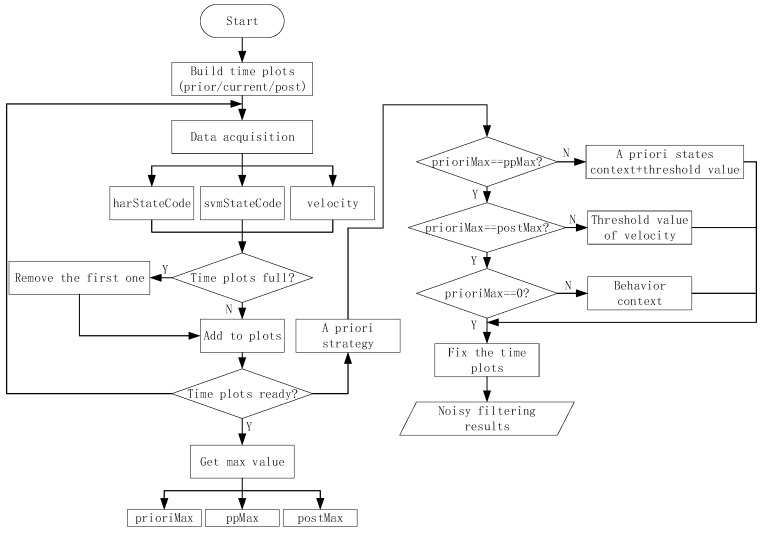
Process flowchart for the time plots.

The state codes are placed into time plots as they are obtained. After the time plots are full, we obtain the voting values that appear the most frequently from each time plot. If there is more than one maximum voting value in a certain time plot, we should consider the a priori context results as the maximum voting values. Then, we use the three maximum voting values obtained from time plots 1–3 to generate the noise filtering result. If these three values are completely different, then we obtain the noise value according to the behavior context, updating the values of the time plots. As shown in Figure 4, we consider a certain state in *BS* as an example. For convenience, we only consider a specific state named A, and any other state is referred to as $A^{-}$. In Figure 5, the maximum voting values obtained from the corresponding time plots are 1, 2, and 3. We combine these three values with the behavior context to obtain the final noise filtering result. There are four circumstances, as follows. In Figure 5a, the three values are the same; they are the specified state. Thus, the result is the same as the values. In Figure 5b, the three values are $A^{-}$. In this case, the values may be completely different; thus, the result depends on the behavior context and is temporarily marked as “?”. In Figure 5c, there is only one state that does not satisfy state A. This state can be considered as noise data and can be removed. In this case, the results are the same as the majority values. In Figure 5d, only one value satisfies state A, and the remaining values may be different. Therefore, we consider that the noise filtering result should be based on the a priori behavior context and the priorities of svmStateCode and harStateCode. Here, we substitute “?” for the noise filtering results in order to further process which will be used into the flowing part of state chain. As shown in Figure 6, it’s a process flowchart of time plots.

#### 2.2.3. Optimization Based on a State Chain

On the basis of the given noise filtering results, we propose an optimization method based on a state chain to reduce the time delay of the basic state events. As indicated in Table 1, these basic state events are <Ca, Wa>, <Ca, Ca>, <Wa, Wa>, and <Wa, Ca>. However, switching to the corresponding positioning algorithm will occur when a user is in <Ca, Wa> or <Wa, Ca>. A few states in <Ca, Ca> or <Wa, Wa> may be stationary or other states, which do not change a user’s localization. In addition, $state_{start}^{i}$ and $state_{end}^{i}$ are the same; hence, we do not change the positioning algorithm. We should recognize the current state precisely and reduce the time delay of each different state as accurately as possible to ensure switching to the correct localization algorithm. Therefore, we use a state chain to further filter the noisy data and optimize the time delay for recognition.

**Figure 7:**
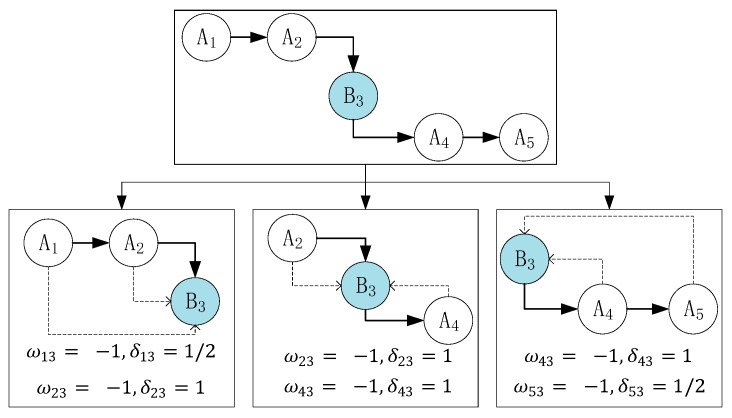
Example of calculating the final influence factor.

We present the concept of a state chain under the condition of coarse time granularity. Considering the relationship between the current-state context and the a priori context/posterior context, we establish a weight $\omega_{ij}$, which indicates the consistency between the two state contexts, and a distance influence factor $\delta_{ij}$, which means farther distance between two states, the less influence the former state has on the latter one. For example, in Figure 7, the distance between $B_{3}$ and $A_{1}$ is 2, so $\delta_{13}=1/2$. And also, $\delta_{23}=1$. We use these factors to model the state influence factor, $I_{j}$, of the state chain. Then, we obtain the chain type to fix it and output the final human activity state. Note that each state chain has 50% overlap with another chain. When the states are the same as the current state, the weight is 1; otherwise, it is −1. $\delta_{ij}$ is inversely proportional to the distance between the *i*-th and *j*-th noise filtering results. The entire state chain contains the following three parts: the a priori context $I_{k}^{1}$, current context $I_{k}^{2}$, and posterior context $I_{k}^{3}$. Each of these contexts is a sum of the products of the weights and influence factors:

(8) $$I_{k}=\;I_{k}^{1}+I_{k}^{2}+I_{k}^{3},$$

where, *k* represents the number index of the middle state in a chain. For instance, in Figure 7, *k* = 3 in that the index of $B_{3}$ is 3:

$$\{\begin{array}{c} I_{k}^{1}=\omega_{k-2,k}\times \delta_{k-2,k}+\omega_{k-1,k}\times \delta_{k-1,k}\;\\ I_{k}^{2}=\omega_{k-1,k}\times \delta_{k-1,k}+\omega_{k+1,k}\times \delta_{k+1,k}\;\\ I_{k}^{3}=\omega_{k+1,k}\times \delta_{k+1,k}+\omega_{k+2,k}\times \delta_{k+2,k} \end{array},\;\left(k\geq 2\right).$$

As shown in Figure 7, we consider the time series A→A→B→A→A as an example. This series can be separated into the following three parts: $I_{3}^{1}$ (A→A→B), $I_{3}^{2}$ (A→B→A), and $I_{3}^{3}$ (B→A→A). Readers may think that the current state $B_{3}$ in the current context is not the actual state and that $A_{5}$ is the actual state at the current time. Here, we consider that if the time interval is considerably small (approximately 2–4 s), then these two states are the same, namely, $B_{3}=A_{5}$. For this equation, there is an inherent relationship that the latter state is influenced by the former state in short time interval, like $B_{3}$ has influence on $A_{5}$. From the first part, the distance between $A_{1}$ and $B_{3}$ is 2; thus, the distance influence factor $\delta_{13}=1/2$. In addition, $A_{1}$ and $B_{3}$ are different states; hence, the weight $\omega_{13}=-1$. The other parameters can be obtained in the same manner. Therefore, the final state influence factor is $I_{3}=I_{3}^{1}+I_{3}^{2}+I_{3}^{3}=\left(-\frac{3}{2}\right)+\left(-2\right)+\left(-\frac{3}{2}\right)=-5$ (here, the subscript number 3 of I comes from $B_{3}$). From Figure 7, we find that the middle state $B_{3}$ is different from other states; thus, we regard this state as noisy data and fix it by replacing it with A.

**Figure 8:**
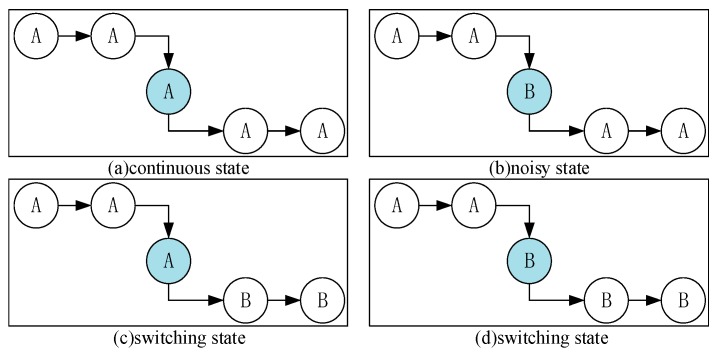
Instances of the three categories.

In the state chain method, we classify all possible state chains into three categories: the continuous, switching, and noisy states. Each state has a unique feature. We use these features to determine the category that a state belongs to. Figure 8 shows a few instances of these categories.

**Figure 9:**
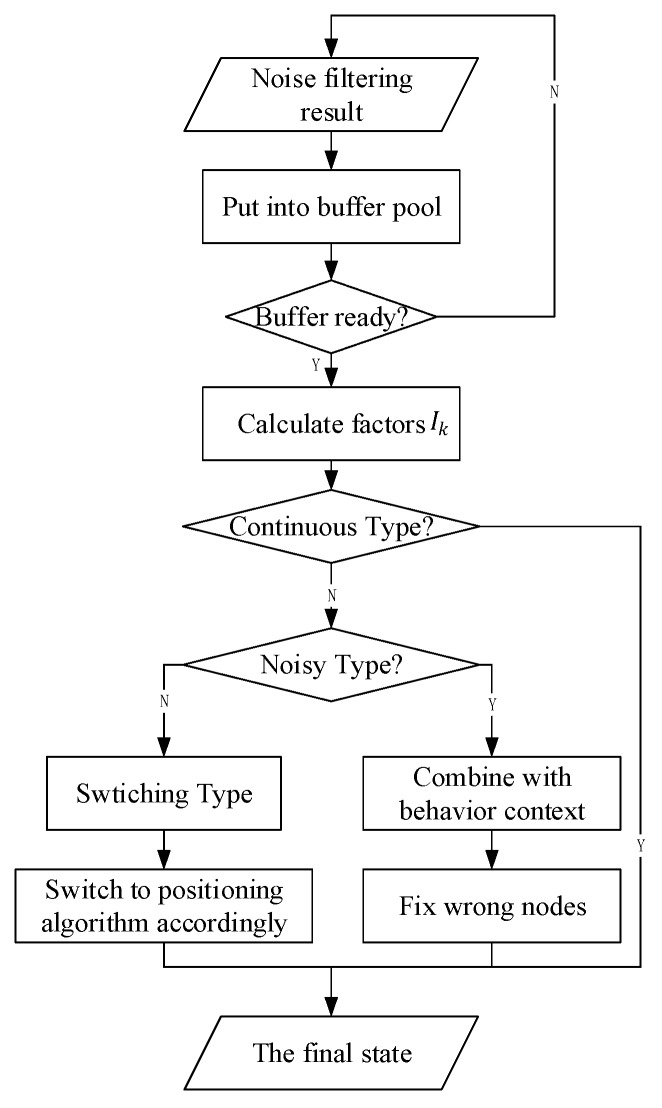
Flowchart of state chain method.

As seen in Figure 8a, there is only one case in the continuous state, and the value of the final influence factor in this case is $\left[\frac{3}{2}+2+\frac{3}{2}\right]=5$. Figure 8c,d show the switching states, and the final influence factor is 0. There are several cases for the noisy state. Figure 8b shows one of them, and the final influence factor is −5. Therefore, we can confirm which category the state chain belongs to, fix the noise on the basis of the behavior context by replacing the state A, and recognize the switching node. Figure 9 shows the flowchart of the state chain method.

After obtaining the final states, we can build end-to-end basic state events such as 〈Ca,Wa〉, 〈Ca,Ca〉,〈Wa,Wa〉, and 〈Wa,Ca〉. If the current states belong to 〈Ca,Ca〉 or 〈Wa,Wa〉, the positioning algorithm is not changed. If they belong to 〈Wa,Ca〉 or 〈Ca,Wa〉, switching to the corresponding algorithm occurs.

## 3. Experiments and Discussion

**Figure 10:**
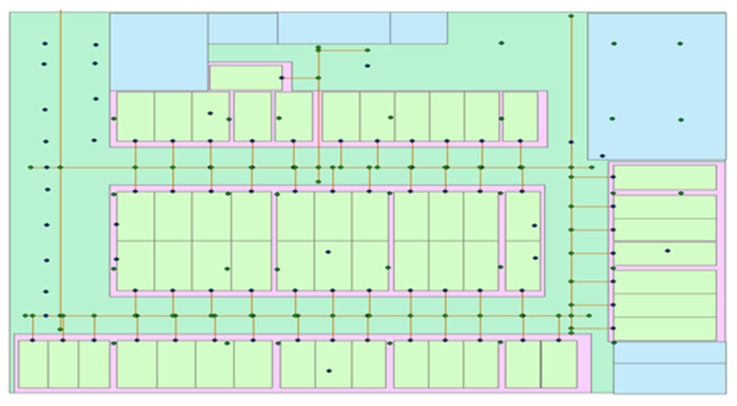
Experimental site.

We performed a few experiments using the proposed method. We selected a parking lot at Wuhan University as the experimental site. Bluetooth devices were deployed and combined with the sensors embedded in mobile phones to realize the switching positioning algorithm. Figure 10 shows the experimental site, and the points show the Bluetooth devices.

We describe the related work on data collection before presenting our experiments. We used two assessment factors to evaluate the proposed method: the rate of correct judgement and the time delay. We aim to recognize each state as accurately as possible so that the entire behavior movement can be more precise. Thus, we use the rate of correct judgement as the factor that represents the accuracy. In addition, we aim to switch to the appropriate positioning algorithm as accurately as possible; hence, we cannot neglect the importance of the time delay. The sensitivity of the method increases as the time delay decreases. Therefore, we tested numerous behavior states. The experiments consisted of the following two parts: a single-behavior-state experiment to evaluate the accuracy of recognition using the rate of correct judgement and a multistate-event experiment with continuous multiple states to assess the influence of the time delay. We can assess the proposed method by acquiring the actual states. On the basis of the experimental results, we analyze the related consequences and discuss the proposed method.

### 3.1. Data Collection

Before recognizing the current motion mode of users, we must collect raw data for various movement states using inertial sensors (e.g., accelerometers and gyroscopes) as the input of deep learning neural networks for training and evaluation. Three-axis (X, Y, Z) data are acquired from the inertial accelerometers and gyroscopes in mobile phones, both of which provide excellent performance in extracting behavior features. The linear accelerated velocity of users can be collected from accelerometers and can be transformed into output signals. Gyroscopes measure the angular velocity related to the deflection and slope. Each movement state has its characteristics in these data. Therefore, we can use these data to analyze the movements of users. On this basis, we analyze the unique features by processing the raw data among the four categories. For the stationary state, the change in the accelerometer values remains smooth. When a user is walking, the acceleration data fluctuate regularly with a certain period. For other states, these data change without any regularity. In addition, when sitting in a car, captured data have different characteristics according to the movement states such as speeding up, slowing down, or braking; however, there is a clear distinction compared to the stationary state in terms of the velocity.

Theoretically, naturalistic environments are desired for our experiments; however, it is not practicable to apply these environments [3]. Thus, we select a specific parking lot to carry out controlled experiments [3]. We consider various situations such as mobile phone locations for different behaviors, which result in differences between the same movements, to collect as many different types of data as possible to include diverse behavior states for training. Table 2 summarizes the various types of data captured, each class declares what kind of data we should collect in order to cover various situations as much as possible.

**Figure 11:**
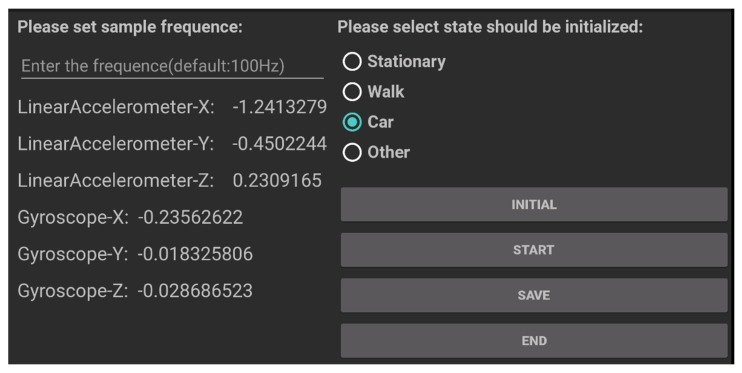
Data collection application.

In the data collection campaign, 10 users carried phones (HUAWEI mate9, XIAOMI NOTE, MEIZU) with them. Each type of data set is at least 40 min, and the interval time is at least 20 s. We developed a simple application for collecting data; it obtains the accelerated velocity from the inertial sensors. The interface is shown in Figure 11.

We preprocess the raw collected data to remove noise using a third-order median filter, a low-pass Butterworth filter with a cutoff frequency of 20 Hz, and a high-pass filter [29]. The collected data are used as the input for CNN training. In addition, we must collect the velocity of a target movement on the basis of Bluetooth positioning. This is because the use of only the results from the CNN cannot ensure highly precise state recognition with 100% certainty. The noise states will appear in the correct behavior state. In addition, when sitting in a car that is moving at a constant speed, it is difficult to distinguish between the in-car and stationary states using only the CNN recognition result. Thus, we cannot fully depend on the CNN result and should utilize these states’ unique features that are different from the others to obtain the correct results. Therefore, we use the difference between the velocities of each state for our proposed method. On this basis, we collected the velocity of a target movement on the basis of Bluetooth positioning to make the localization as accurate as possible. Given the precise position coordinates, $\left(x_{t},y_{t}\right)$ and $\left(x_{t+interval},y_{t+interval}\right)$, we can calculate a target’s velocity as:

(9) $$velocity=\;\sqrt{{\left(x_{t+interval}-x_{t}\right)}^{2}\;+\;{\left(y_{t+interval}-y_{t}\right)}^{2}}/interval,$$

where $\left(x_{t},y_{t}\right)$ are the coordinates at time t, and interval is the time interval. The velocity is the average speed within a specified time period. Thus, we can utilize the given initial state recognition results and speed data to solve problems.

### 3.2. Assessment Factors

As stated earlier, we use two factors for the experimental assessment. The first is the rate of correct judgment $R_{cor}$, which indicates the recognition accuracy of the states, and the second is the time delay, which are respectively expressed as:

(10) $$R_{cor}=N_{hit}/N_{total},$$

(11) $$t_{delay}=t_{exper}-t_{real}.$$

In a certain time sequence, the behavior state results are output every few seconds. There are error states among these results, and we use the rate of correct judgment to assess the accuracy of each state in BS. $N_{total}$ is the total number of results output in a time sequence. $N_{hit}$ is the number of correct results. When $N_{total}$ remains unchanged, $R_{cor}$ increases with $N_{hit}$. $T_{delay}$ is used to measure the time delay by comparing the actual behavior context and the experimental results. $T_{exper}$ is the time required to change between different states during experiments. $t_{real}$ is the time in the actual situation. If $t_{exper}\to t_{real}$, then $t_{delay}\to 0$.

### 3.3. Single-Behavior-State Experiment

**Figure 12:**
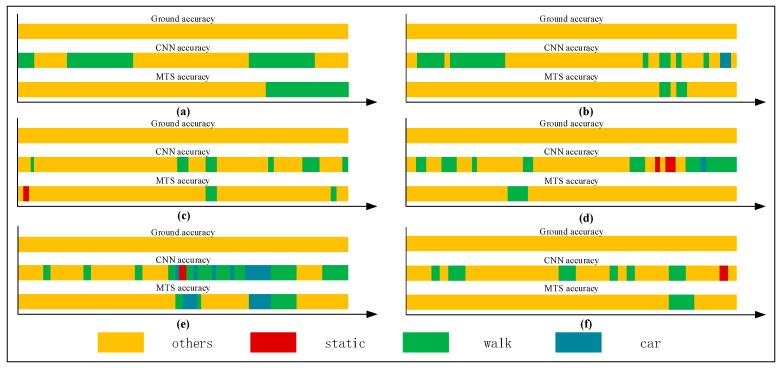
Graphs showing recognition of the other states.

**Figure 13:**
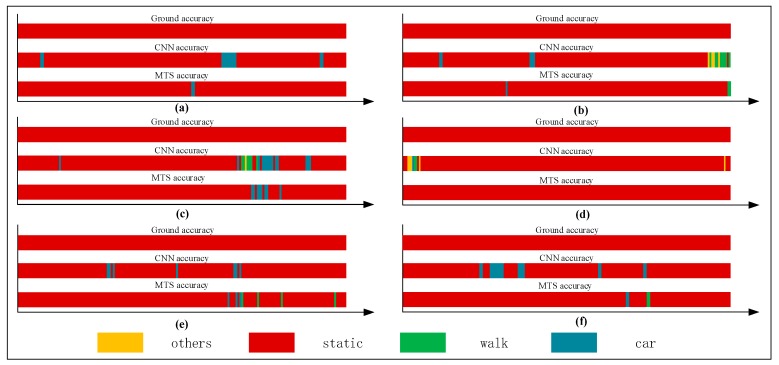
Graphs showing recognition of the stationary state.

**Figure 14:**
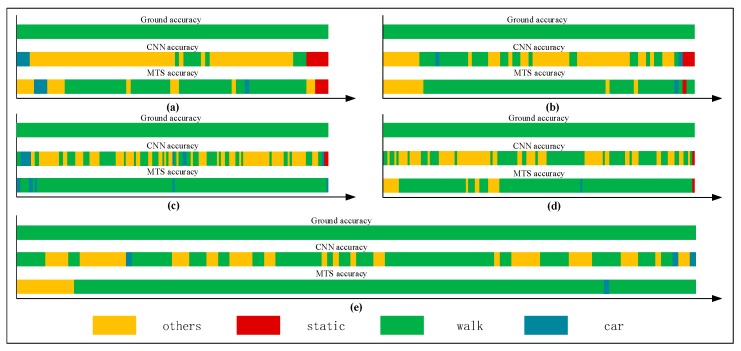
Graphs showing recognition of the walking state.

**Figure 15:**
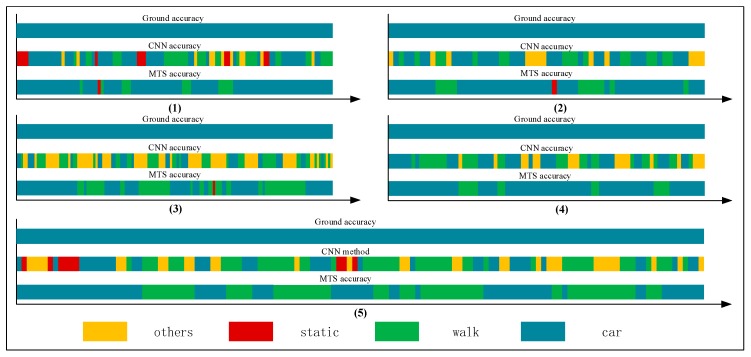
Graphs showing recognition of the in-car state.

For a single human movement state, we mainly use the single-behavior-state experiment to assess the recognition effect adopting the rate of correct judgment. Table 3, Table 4, Table 5 and Table 6 list comparisons of group tests using the CNN and proposed methods. For each tabulated index, the respective graphs of the results are shown in Figure 12, Figure 13, Figure 14 and Figure 15.

We collected BS for the stationary, others, walking, and in-car states separately. Each certain state can output the current result every few seconds. When the generated states are almost the same as those obtained for the real result, $N_{hit}\to N_{total}$, and $R_{cor}$ is closer to 1; this is the best result obtained theoretically.

Figure 12, Figure 13, Figure 14 and Figure 15 show that the upper, middle and bottom bands are a real situation named ground accuracy, the result of using CNN method, only named CNN accuracy, and the result of using the improved method is called MTS accuracy, respectively. For the other states, it is easy to generate noisy data in the walking state. The results are improved by approximately 21% on average in Figure 12. For each group of experiments and for the same value of $N_{total}$, compared with the CNN accuracy, the result of MTS accuracy was considerably improved from approximately 10.34% to at least 31.11%. For the static state, owing to its apparent and unique features that are different from those of other states, the effect of only using the CNN method is acceptable. When we employed the proposed adaptive method, the obtained result is better by approximately 5.5% on average, but the consequence of walking is increased by approximately 45.47% on average. In addition, the accuracy of the in-car state also increases by approximately 26.23%.

**Figure 16:**
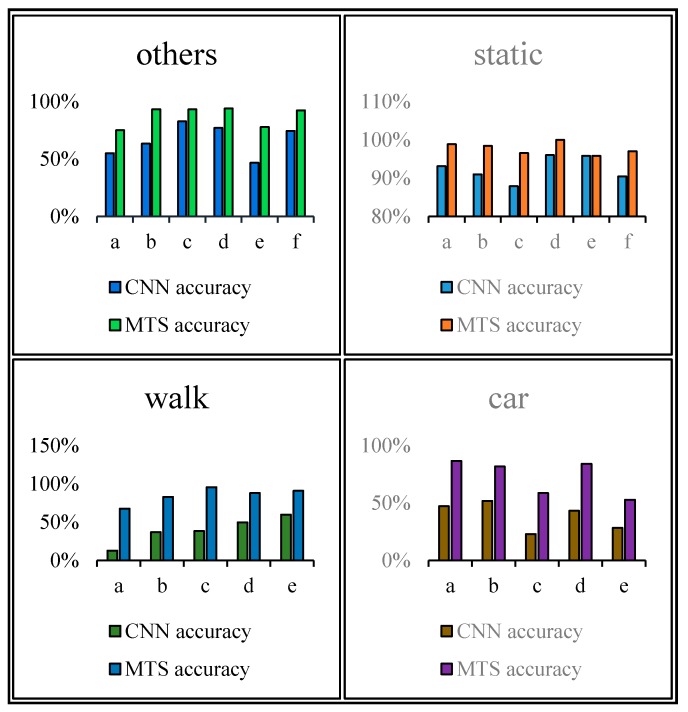
Comparison of the CNN and MTS accuracies.

Figure 16 shows a comparison of the results obtained using the CNN and proposed method. As shown, all single states can be recognized more accurately when using the proposed method. In particular, the walking and in-car state recognition is improved considerably.

### 3.4. Multistate-Event Experiment

**Figure 17:**
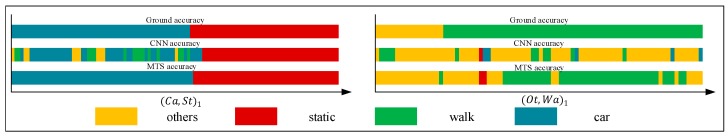
Graphs of ${\langle Ca:Wa\rangle }_{1}$.

**Figure 18:**
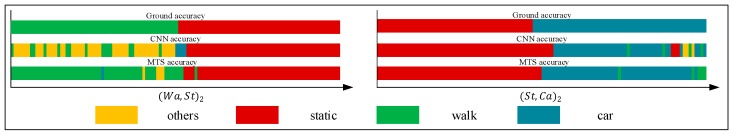
Graphs of ${\langle Wa:Ca\rangle }_{2}$.

**Figure 19:**
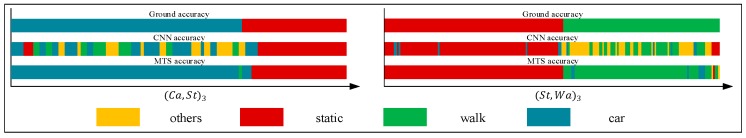
Graphs of ${\langle Ca,Wa\rangle }_{3}$.

**Figure 20:**
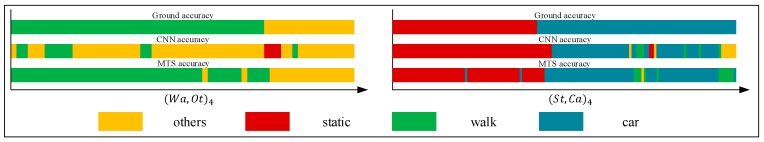
Graphs of ${\langle Wa,Ca\rangle }_{4}$.

**Figure 21:**
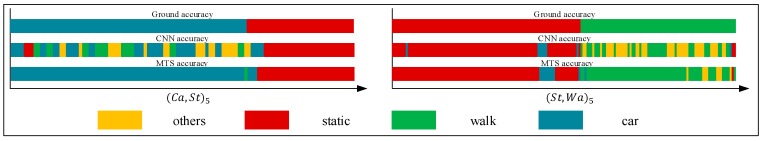
Graphs of ${\langle Ca,Wa\rangle }_{5}$.

In this study, we also performed a multistate-event experiment to evaluate the time delay when switching to the correct positioning algorithm. Tables 8–12 list comparisons of the group tests conducted using the CNN and proposed methods. The respective results for the tabulated indices are displayed in the graphs in Figure 17, Figure 18, Figure 19, Figure 20 and Figure 21.

On the basis of the single-behavior-state experiment, we thoroughly analyzed the multistate events in the multi-event experiment. For a series of complex behavior states, we should detect a change in a state and determine whether it is necessary to switch to the corresponding positioning algorithm. Note that the time interval of the change in the state events should be as short as possible, considering that the recognition accuracy of each state is reliable. In other words, in this situation, we adopt $R_{cor}$ as the estimate of a state and use $t_{delay}$ to measure the sensitivity of switching between different localization algorithms. Therefore, we gathered many state events, as summarized in Table 7 (units: s).

We collected a series of states, as shown in Table 8, Table 9, Table 10, Table 11 and Table 12. The upper, middle, and bottom ribbons in Figure 17, Figure 18, Figure 19, Figure 20 and Figure 21 represent a real situation, the result of using the CNN method only, and the result of using the improved method, respectively. From these, we need to recognize when the trilateral Bluetooth positioning method for the in-car state will be changed and when the PDR positioning algorithm for walking will be switched. These states are aimed at users in locomotion. If there is no movement indoors, the positioning method does not need to be changed. For example, if the state changes from *St* to *Ot*, the user position will not be changed. Thus, the time delay for recognition between *St* and *Ot* is inconsequential. Although the delay time for recognition between *Wa* and *Ca* is significant, we do not need to consider it because in an actual situation, when people are in a car (walking), they cannot change their state to walking (in-car) instantly. That is, after staying in the car, they must be in some other states including the stationary and other states. Thus, we only considered the time delay of some states such as (Ca,St), (Ot,Wa), (Ca,Ot), (Ot,Ca), (St,Wa), (St,Ca), (Wa,Ot), and (Wa,St) but not (Ot,St) and (St,Ot). As indicated in Table 7, we separated *Ca*—*St*—*Ot*—*Wa*—*St*—*Ca*—*St*—*Wa*—*Ot*—*St*—*Ca*—*St*—*Wa* into five state events:$\;{\langle Ca:Wa\rangle }_{1}$, ${\langle Wa:Ca\rangle }_{2}$, ${\langle Ca,Wa\rangle }_{3}$, ${\langle Wa,Ca\rangle }_{4}$, and ${\langle Ca,Wa\rangle }_{5}$, each of which is composed of some basic state events. For instance, ${\langle Ca:Wa\rangle }_{1}$ can be expressed as ${\left(Ca,St\right)}_{1}\cup {\left(St,Ot\right)}_{1}\cup {\left(Ot,Wa\right)}_{1}$. However, according to the abovementioned statement, we only focused on ${\left(Ca,St\right)}_{1}\cup {\left(St,Ot\right)}_{1}\cup {\left(Ot,Wa\right)}_{1}$ and not ${\left(St,Ot\right)}_{1}$. These states play an important role in determining the accuracy of switching between different algorithms. As indicated in Table 3, Table 4, Table 5 and Table 6, the recognition result obtained using only the CNN method is inferior to that obtained using the proposed method.

**Figure 22:**
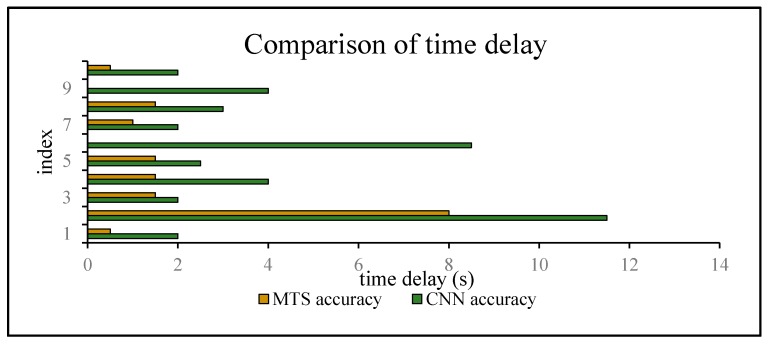
Comparison of the CNN and MTS accuracies with regards to the time delay.

The time delay can be increased by 0.5–8.5 s. For switching between Ca and St, the time delays were reduced by 1.5, 2.5, 1, 1.5, and 1.5 s, in order of the five state events, corresponding to decreases of 75%, 62.5%, 40%, 50%, and 100%, respectively. For Ot and Wa, compared with the CNN method, the time delay was reduced by approximately 40.2175% on average, whereas it can be reduced by approximately 66.67% on average for switching between Wa and St. The graphs show that a considerable amount of noisy data was removed and fixed, and the time delay was apparently shortened. Similarly, Figure 22 shows a comparison of the CNN and MTS accuracies with regards to the time delay. For ${\left(St,Wa\right)}_{3}$, ${\left(St,Wa\right)}_{5}$, and ${\left(St,Ca\right)}_{4}$, the results are improved considerably by approximately 1.5 s and by at least 7 s at maximum compared to the CNN method. As there is an obvious distinction between the stationary and in-car (walking) states, we can easily differentiate between them on the basis of the velocity of the user’s movement and by using step estimation and the step length from the embedded sensors. In addition, the improvement in the time delay for ${\left(Ca,St\right)}_{1}$, ${\left(St,Ca\right)}_{2}$, and ${\left(Ca,St\right)}_{5}$ also has better effects. Although the time delay of some events was shortened by a few seconds, for the entire behavior movement, a large amount of the total time delay can be saved, which can improve the robustness of the algorithm. In our experiment, the time delay was reduced by approximately 24 s over the entire movement with multiple states, even though for each basic state event, the time delay time was optimized by approximately 0.5–8.5 s.

## 4. Conclusions

This paper presented an adaptive method for a switching indoor positioning algorithm based on multilayer time sequences. The method focused on filtering noisy data and shortening the time delay on different scales. To filter noisy data, we adopted three continuous time plots by using the maximum vote rule to fix the data. In addition, we employed a state chain to reduce the time delay and filter noisy data. These two factors make full use of the features of each state to obtain a better result. The in-car and walking states show obvious distinctions in the velocity and step estimation. The velocity of a car is higher than that in the stationary state, whose velocity is close to 0. If a user is in the other state, his/her location will not be changed, and the step estimate cannot be carried out

According to the experiments conducted, the proposed method, combining the CNN method and multilayer time sequences, outperformed the CNN method. The accuracies of the states were improved by 21%, 5.5%, 45.47%, and 26.23% which correspond to “other”, stationary, walking and in-car states. For the stationary state, which is already recognized well using only the CNN method, the result obtained using the proposed method was improved by 5.5%. In addition, the time delay is reduced by approximately 24 s in the entire movement with multiple different states, even though for each basic state event, the time delay time was optimized by approximately 0.5–8.5 s. Although the proposed method considerably improved the results, we still cannot avoid the existence of noisy data and remove the time delay completely. Thus, more research is required to improve using previous related methods as a basis. 

## Figures and Tables

**Table 1:** Basic state events.

Basic State Event ${\langle state_{start}^{i}\;:\;state_{end}^{i}\rangle }_{i}$
<Ca,Wa>
<Ca,Ca>
<Wa,Wa>
<Wa,Ca>

**Table 2:** Data collection types.

First Class (Categories)	Second Class (Location)	Third Class (Action)	Fourth Class (Additional)
Static	Everywhere	Place Hold reading Hold calling In car (constant speed)	None
Walking	Hold in hand	Shaking arm Reading/calling	None
	Place in pocket	In coat In trousers	None
In car	In car	Hold reading Hold calling Place	Speed up/slow down/braking
Others	In bag/hold	Shaking the phone	None

**Table 3:** Recognition results for the other states.

Index	CNN Method Only			MTS Method		
	$N_{hit}$	$N_{total}$	$R_{cor}$	$N_{hit}$	$N_{total}$	$R_{cor}$
a	11	20	55%	15	20	75%
b	38	60	63.33%	56	60	93.33%
c	48	58	82.76%	54	58	93.10%
d	50	65	76.92%	61	65	93.85%
e	42	90	46.67%	70	90	77.78%
f	29	39	74.36%	36	39	92.31%

**Table 4:** Recognition results for the stationary state.

Index	CNN Method Only			MTS Method		
	$N_{hit}$	$N_{total}$	$R_{cor}$	$N_{hit}$	$N_{total}$	$R_{cor}$
a	81	87	93.10%	86	87	98.85%
b	171	188	90.96%	185	188	98.40%
c	152	173	87.86%	167	173	96.53%
d	193	201	96.02%	201	201	100%
e	159	166	95.78%	159	166	95.78%
f	85	94	90.43%	92	94	97.87%

**Table 5:** Recognition results for the walking state.

Index	CNN Method Only			MTS Method		
	$N_{hit}$	$N_{total}$	$R_{cor}$	$N_{hit}$	$N_{total}$	$R_{cor}$
a	9	71	12.67%	48	71	67.61%
b	28	76	36.84%	63	76	82.89%
c	59	154	38.31%	147	154	95.46%
d	69	139	49.64%	122	139	87.77%
e	71	119	59.66%	108	119	90.76%

**Table 6:** Recognition results for the in-car state.

Index	CNN Method Only			MTS Method		
	$N_{hit}$	$N_{total}$	$R_{cor}$	$N_{hit}$	$N_{total}$	$R_{cor}$
a	49	104	47.12%	90	104	86.54%
b	31	60	51.67%	49	60	81.67%
c	32	140	22.86%	82	140	58.57%
d	35	81	43.21%	68	81	83.95%
e	37	131	28.24%	69	131	52.67%

**Table 7:** Multistate-event experiment.

An Example of Locomotion	
Ca→St→Ot→Wa→St→Ca→St→Wa→Ot→St→Ca→St→Wa	
SE	${\langle Ca:Wa\rangle }_{1}\cup {\langle Wa:Ca\rangle }_{2}\cup {\langle Ca:Wa\rangle }_{3}\cup {\langle Wa:Ca\rangle }_{4}\cup {\langle Ca:Wa\rangle }_{5}$
Basic SE	$\;{\langle Ca:Wa\rangle }_{1}={\left(Ca,St\right)}_{1}\cup {\left(St,Ot\right)}_{1}\cup {\left(Ot,Wa\right)}_{1}$
	$\;{\langle Wa:Ca\rangle }_{2}={\left(Wa,St\right)}_{2}\cup {\left(St,Ca\right)}_{2}$
	$\;{\langle Ca,Wa\rangle }_{3}={\left(Ca,St\right)}_{3}\cup {\left(St,Wa\right)}_{3}$
	$\;{\langle Wa,Ca\rangle }_{4}={\left(Wa,Ot\right)}_{4}\cup {\left(Ot,St\right)}_{4}\cup {\left(St,Ca\right)}_{4}$
	$\;{\langle Ca,Wa\rangle }_{5}={\left(Ca,St\right)}_{5}\cup {\left(St,Wa\right)}_{5}$

**Table 8:** Basic SE of ${\langle Ca:Wa\rangle }_{1}$.

Event Nodes	Index	CNN Mthod Only			MTS Method		
		$t_{exper}$	$t_{real}$	$t_{delay}$	$t_{exper}$	$t_{real}$	$t_{delay}$
${\left(Ca,St\right)}_{1}$	1	31.5	29.5	2	30	29.5	0.5
${\left(Ot,Wa\right)}_{1}$	2	20	8.5	11.5	16.5	8.5	8

**Table 9:** Basic SE of ${\langle Wa:Ca\rangle }_{2}$.

Event Nodes	Index	CNN Method Only			MTS Method		
		$t_{exper}$	$t_{real}$	$t_{delay}$	$t_{exper}$	$t_{real}$	$t_{delay}$
${\left(Wa,St\right)}_{2}$	3	32.5	30.5	2	32	30.5	1.5
${\left(St,Ca\right)}_{2}$	4	30.5	26.5	4	28	26.5	1.5

**Table 10:** Basic SE of ${\langle Ca,Wa\rangle }_{3}$.

Event Nodes	Index	CNN Method Only			MTS Method		
		$t_{exper}$	$t_{real}$	$t_{delay}$	$t_{exper}$	$t_{real}$	$t_{delay}$
${\left(Ca,St\right)}_{3}$	5	39	36.5	2.5	38	36.5	1.5
$\;{\left(St,Wa\right)}_{3}$	6	63.5	55	8.5	55	55	0

**Table 11:** Basic SE of ${\langle Wa,Ca\rangle }_{4}$.

Event Nodes	Index	CNN Method Only			Proposed Method		
		$t_{exper}$	$t_{real}$	$t_{delay}$	$t_{exper}$	$t_{real}$	$t_{delay}$
${\left(Wa,Ot\right)}_{4}$	7	24.5	22.5	2	23.5	22.5	1
${\left(St,Ca\right)}_{4}$	8	32	29	3	30.5	29	1.5

**Table 12:** Basic SE of ${\langle Ca,Wa\rangle }_{5}$.

Event Nodes	Index	CNN Method Only			Proposed Method		
		$t_{exper}$	$t_{real}$	$t_{delay}$	$t_{exper}$	$t_{real}$	$t_{delay}$
${\left(Ca,St\right)}_{5}$	9	41.5	37.5	4	37.5	37.5	0
${\left(St,Wa\right)}_{5}$	10	50	48	2	48.5	48	0.5

## References

[1] Xu R., Chen W., Xu Y., Ji S., Liu J. (2017). Improved GNSS-based indoor positioning algorithm for mobile devices. GPS Solut..

[2] Retscher G., Tatschi T. Indoor Positioning Using Wi-Fi Lateration—Comparison of Two Common Range Conversion Models with Two Novel Differential Approaches. Proceedings of the 2016 Fourth International Conference on Ubiquitous Positioning, Indoor Navigation and Location Based Services (UPINLBS).

[3] Zou H., Jiang H., Luo Y., Zhu J., Lu X., Xie L. (2016). BlueDetect: An iBeacon-enabled scheme for accurate and energy-efficient indoor-outdoor detection and seamless location-based service. Sensors.

[4] Vahidnia M.H., Malek M.R., Mohammadi N., Alesheikh A.A. (2013). A hierarchical signal-space partitioning technique for indoor positioning with WLAN to support location-awareness in mobile map services. Wirel. Pers. Commun..

[5] Bahl P., Padmanabhan V.N. RADAR: An In-Building RF-based User Location and Tracking System. Proceedings of the IEEE INFOCOM 2000 Nineteenth Annual Joint Conference of the IEEE Computer and Communications Societies.

[6] Chen X., Zou S. (2017). Improved Wi-Fi indoor positioning based on particle swarm optimization. IEEE Sens. J..

[7] Caso G., De Nardis L. (2016). Virtual and oriented WiFi fingerprinting indoor positioning based on multi-wall multi-floor propagation models. Mobile Netw. Appl..

[8] Chen Y., Xue Y. A Deep Learning Approach to Human Activity Recognition Based on Single Accelerometer. Proceedings of the IEEE International Conference on Systems.

[9] Ma Z., Poslad S., Bigham J., Zhang X., Men L. A BLE RSSI Ranking based Indoor Positioning System for Generic Smartphones. Proceedings of the Wireless Telecommunications Symposium.

[10] Retscher G. (2017). Fusion of location fingerprinting and trilateration based on the example of differential Wi-Fi positioning. ISPRS Ann. Photogramm. Remote Sens. Spatial Inf. Sci..

[11] Zhou C., Yuan J., Liu H., Qiu J. (2017). Bluetooth indoor positioning based on RSSI and Kalman filter. Wirel. Pers. Commun..

[12] Lee M.S., Ju H., Park C.G. (2017). Map assisted PDR/Wi-Fi fusion for indoor positioning using smartphone. Int. J. Control Autom. Syst..

[13] iBeacon for Developers. https://developer.apple.com/ibeacon/.

[14] Ravi D., Wong C., Lo B., Yang G.-Z. Deep Learning for Human Activity Recognition: A Resource Efficient Implementation on Low-Power Devices. Proceedings of the IEEE 13th International Conference on Wearable and Implantable Body Sensor Networks.

[15] Vieira M.A., Vieira M., Louro P., Mateus L., Vieira P. (2016). Indoor positioning system using a WDM device based on a-SiC: H technology. J. Lumin..

[16] De Blasio G., Quesada-Arencibia A., Garcia C.R., Molina-Gil J.M., Caballero-Gil C. (2017). Study on an indoor positioning system for harsh environments based on Wi-Fi and Bluetooth Low Energy. Sensors.

[17] Altun K., Barshan B., Tunel O. (2010). Comparative study on classifying human activities with miniature inertial and magnetic sensors. Pattern Recognit..

[18] Pratama A.R., Hidayat R. Smartphone-based Pedestrian Dead Reckoning as an Indoor Positioning System. Proceedings of the International Conference on System Engineering and Technology.

[19] Groves P.D., Martin H., Voutsis K., Walter D., Wang L. Context Detection, Categorization and Connectivity for Advanced Adaptive Integrated Navigation. Proceedings of the International Technical Meeting of the Satellite Division of the Institute of Navigation.

[20] Vaizman Y., Ellis K., Lanckriet G. (2017). Recognizing detailed human context in the wild from smartphones and smartwatches. IEEE Pervasive Comput..

[21] Gao H., Groves P.D. Context Determination for Adaptive Navigation using Multiple Sensors on a Smartphone. Proceedings of the 29th International Technical Meeting of The Satellite Division of the Institute of Navigation (ION GNSS+ 2016).

[22] Li H., Wei D., Lai Q., Zu Y., Yuan H. (2017). Smartphone-based integrated PDR/GPS/Bluetooth pedestrian location. Adv. Space Res..

[23] Parviainen J., Bojja J., Collin J., Leppänen J., Eronen A. (2014). Adaptive activity and environment recognition for mobile phones. Sensors.

[24] Pei L., Liu J., Guinness R., Chen Y., Kuusniemei H., Chen R. (2012). Using LS-SVM based motion recognition for smartphone indoor wireless positioning. Sensors.

[25] Kavanagh J.J., Menz H.B. (2008). Accelerometry: A technique for quantifying movement patterns during walking. Gait Posture.

[26] Xu Y., Shen Z., Zhang X., Gao Y., Deng S., Wang Y., Fan Y., Chang E.I. (2017). Learning multi-level features for sensor-based human action recognition. Pervasive Mob. Comput..

[27] Gu T., Wang L., Chen H., Tao X., Lu J. (2011). Recognizing multiuser activities using wireless body sensor networks. IEEE Trans. Mob. Comput..

[28] Zwirello L., Schipper T., Harter M., Zwick T. (2012). UWB localization system for indoor applications: Concept, realization and analysis. J. Elect. Comput. Eng..

[29] Ortiz J.L.R. (2015). Smartphone-Based Human Activity Recognition.

[30] Micucci D., Mobilio M., Napoletano P. (2017). UniMiB SHAR: A dataset for human activity recognition using acceleration data from smartphones. Appl. Sci..

[31] Lee S.-M., Yoon S.M., Cho H. Human Activity Recognition from Accelerometer Data Using Convolutional Neural Network. Proceedings of the IEEE International Conference on Big Data and Smart Computing.

[32] Gjoreski H., Bizjak J., Gjoreski M., Gams M. Comparing Deep and Classical Machine Learning Methods for Human Activity Recognition using Wrist Accelerometer. Proceedings of the IJCAI 2016 Workshop on Deep Learning for Artificial Intelligence.

[33] Susi M., Borio D., Lachapelle G. Accelerometer Signal Features and Classification Algorithms for Positioning Applications. Proceedings of the International Technical Meeting of the Institute of Navigation.

[34] Kraemer I., Eissfeller B. A-GNSS: A Different Approach. http://www.insidegnss.com/node/1636.

[35] Frank K., Vera Nadales M.J., Robertson P., Angermann M. Reliable Real-Time Recognition of Motion Related Human Activities Using MEMS Inertial Sensors. Proceedings of the 23rd International Technical Meeting of The Satellite Division of the Institute of Navigation (ION GNSS 2010).

[36] Elhoushi M., Georgy J., Noureldin A., Korenberg M.J. (2015). Motion mode recognition for indoor pedestrian navigation using portable devices. IEEE Trans. Instrum. Meas..

